# Myocardial radiomics of non-ischemic cardiomyopathy using cardiovascular magnetic resonance: current perspectives and future directions

**DOI:** 10.3389/fcvm.2026.1637962

**Published:** 2026-02-11

**Authors:** Samir Zaman, Prabhu Sasankan, Amine Amyar, Connie W. Tsao

**Affiliations:** Cardiovascular Division, Department of Medicine, Beth Israel Deaconess Medical Center, Harvard Medical School, Boston, MA, United States

**Keywords:** artificial intelligence, cardiovascular magnetic resonance, machine learning, non-ischemic cardiomyopathy, radiomics

## Abstract

Heart failure remains a major source of global morbidity and mortality, frequently driven by the structural and functional myocardial changes associated with ischemic and non-ischemic cardiomyopathies. While cardiovascular magnetic resonance (CMR) is the gold standard for non-invasive ventricular assessment, standard clinical measures rely on visual human interpretation. By contrast, radiomic analysis, a high-throughput computational approach that can extract quantitative features beyond the limits of visual perception, has gained interest in its application to CMR for detailed evaluation of myocardial properties. Over the last decade, novel studies integrating radiomics with machine learning (ML) algorithms may enable more accurate diagnosis and personalized characterization of non-ischemic cardiomyopathy beyond traditional CMR sequences, and without the use of gadolinium-based contrast agents. This review provides an overview of CMR radiomic analysis, summarizes recent applications of ML workflows in non-ischemic cardiomyopathy, and discusses the challenges and opportunities in integrating these computational tools into clinical practice.

## 1 Introduction

Over 6 million individuals in the U.S. are diagnosed with heart failure and over 55 million individuals are diagnosed worldwide (1). Ischemic and non-ischemic cardiomyopathies are characterized by abnormalities in cardiac structure and function and frequently underlie heart failure. Presently, cardiovascular magnetic resonance imaging (CMR) is the gold standard non-invasive imaging method to evaluate for these abnormalities (2). CMR cine sequences can be used for qualitative or quantitative measures of ventricular structure and function including left ventricular (LV) mass, volume, or myocardial scarring; moreover, administration of gadolinium-based contrast agents in CMR sequences has been used to improve myocardial tissue characterization via gadolinium enhanced imaging. More recently, the use of parametric mapping, which generates a pixel-wise map of magnetic resonance properties of tissue (3), has enabled deeper characterization of the myocardium. Structural and tissue-level characterization of the myocardium and its pathology is particularly valuable in the evaluation of diffuse myocardial disease that is often characteristic of non-ischemic cardiomyopathy (NICM).

Radiomic analysis, which leverages advanced analysis of pixel- and voxel-level patterns of an image to extract a vast array of features, is a burgeoning field that enables more detailed and objective characterization of tissue structure and properties from medical images. Pioneered in the field of oncology where it has led to significant advances in tumor characterization (4), radiomics has numerous applications in cardiac imaging. Applied to CMR, radiomics can derive features of cardiac shape, myocardial texture, intensity, and higher-order statistics of pixel variations beyond traditional qualitative assessments or limited quantitative measures of ventricular volume and mass. It offers a particular promise in the diagnosis and evaluation of NICM, where tissue heterogeneity, fibrosis, and other diffuse abnormalities often elude conventional imaging assessment.

Advances in machine learning (ML) have enabled advanced computational image analysis to extend the capabilities of CMR in the diagnosis and characterization of NICM. Applying ML algorithms in the analysis of radiomic data offers a potential avenue to enhance diagnostic accuracy, prognostication, and treatment stratification for numerous forms and etiologies of NICM. In this review, we will provide an overview of the routinely acquired CMR sequences available for radiomic analysis, review its applications in the evaluation of NICM, and suggest areas for further opportunity in this field.

## 2 Clinical CMR sequences for characterization of cardiac structure and function

Non-invasive characterization of myocardial structure and function is an important step for determining the underlying etiology of a cardiomyopathy. Moreover, repeated evaluation is key to monitoring response to treatment and risk stratification for future complications like ventricular arrhythmia. CMR for cardiomyopathy includes T1-weighted, T2-weighted, and gadolinium-contrast enhanced cine imaging sequences as well as late gadolinium enhancement (LGE) imaging, well-regarded as the gold standards for the assessment fibrosis, edema, and infiltrative disease (5–7). However, traditionally, these modalities rely on visual assessment of signal intensity, thus limiting the ability to assess myocardial pathology that is uniform throughout the myocardium, such as diffuse myocardial inflammation, fibrosis, hypertrophy, or infiltration (8). In recent years, rapid parametric mapping techniques such as T1, T2, and T2* mapping, which yield quantitative data that captures various tissue-specific properties of the myocardium, have broadened the scope of tissue characterization and the ability for histologic inference (9, 10). Compared to T2 and T2*, T1 mapping has been the most widely used and broadly applicable for radiomic analysis, as well as the focus of many studies utilizing ML algorithms in radiomic data analysis.

T1 mapping generates a pixel-wise map of T1 relaxation times, an inherent property of the tissue reflecting its composition. Native T1 mapping, performed in the absence of GBCA, reflects the condition of both myocytes and the interstitial spaces, allowing for detection of myocardial abnormalities such as edema, protein deposition, and iron overload. Post-contrast T1 mapping, performed with GBCA, allows for an estimation of the extracellular volume (ECV) which aids in quantification interstitial fibrosis and infiltration. Thus, T1 mapping offers advantages in characterizing myocardial infarction, myocarditis, and systemic diseases such as amyloidosis and Fabry disease.

### 2.1 Radiomics and artificial intelligence (AI)

**Figure 1:**
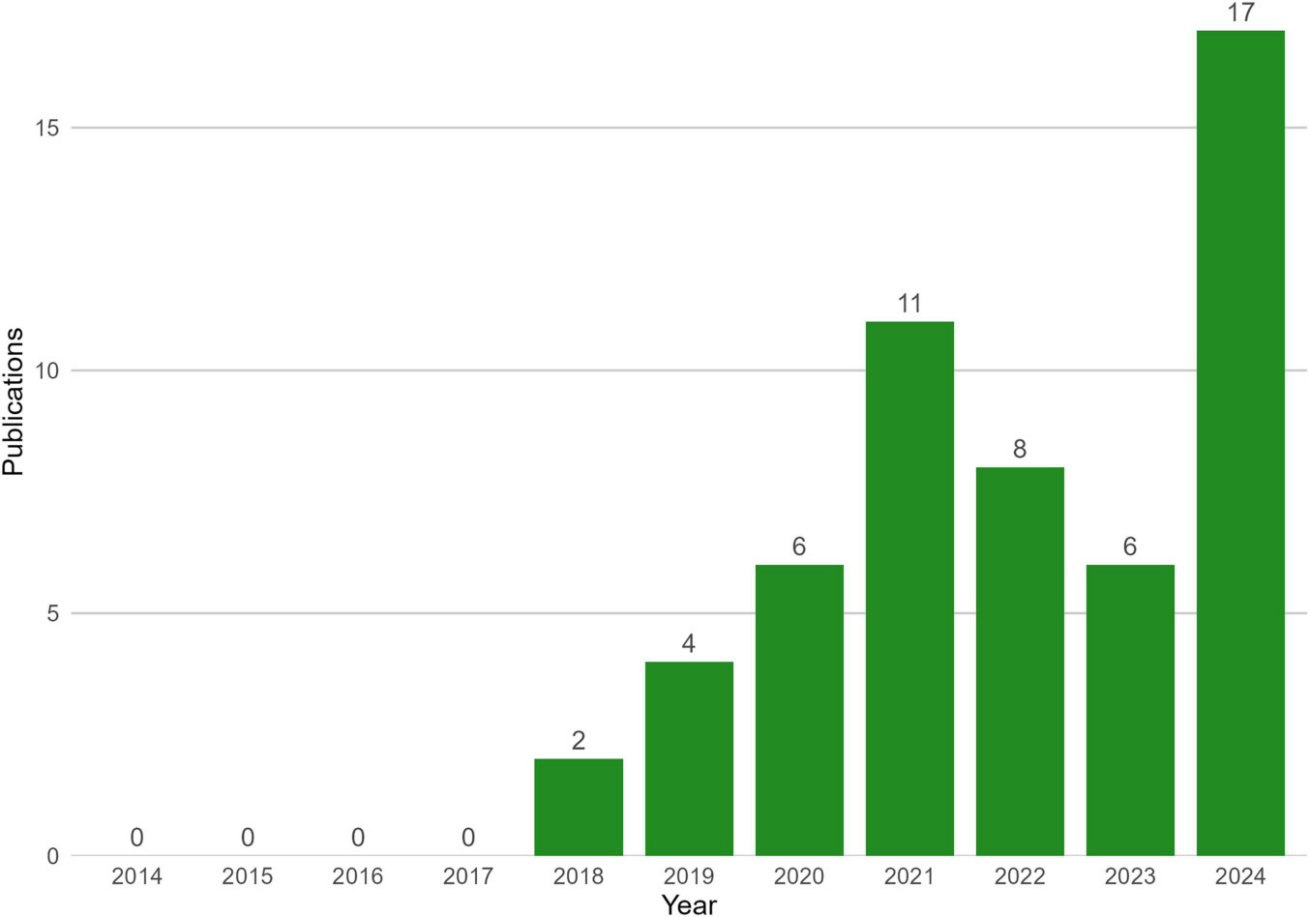
CMR radiomics publications by year. Using PubMed database searching for publications with search strategy for “Radiomics” AND “cardiac magnetic resonance imaging” via MeSH was conducted on 05/04/2025. Publications between 2014 and 2024 are shown.

Radiomics allows for the extraction and analysis of quantitative features from medical images, including texture, intensity, and shape. This approach transforms standard image sequences into rich, mineable datasets to uncover patterns and insights otherwise imperceptible to the unassisted eye (11). There is a growing interest in applying radiomic techniques to CMR (Figure 1), and a particular enthusiasm for its potential to help better differentiate cardiomyopathies. Presently, traditional classification systems may not adequately capture the pathologic diversity between cardiomyopathies. Numerous cardiomyopathies with varying management and prognoses have overlapping features across multiple modalities of non-invasive imaging. Recent radiomic advances may uncover unique imaging phenotypes associated with specific diseases, including even genetic or molecular profiles (12–16). Continued advancement in radiomic analysis will lead to expansion in the scope of tissue characterization and improvement in our ability to distinguish different disease states. Moreover, these previously unappreciated features of analysis may lead to evolution of our very definitions of these diseases. Increasingly, AI techniques, particularly ML, are being leveraged to develop predictive models from radiomic data, further enhancing its clinical utility (17). These advances additionally improve the depth of analysis from non-contrast CMR sequences. As radiomics analysis continues to develop, this may lead to a reduced need for gadolinium contrast, reducing risk of associated toxicity as well as healthcare costs (18–20).

### 2.2 Radiomics features

Broad myocardial radiomics feature groups assessed include shape, statistical, and texture features (Table 1). Shape features quantify the geometric properties of the myocardium, including volume, surface area, compactness, and sphericity. These features may capture global structural alterations in the heart associated with pathological conditions, including LV hypertrophy, dilated cardiomyopathy (DCM), and right ventricular dilation secondary to pulmonary hypertension. First-order features quantify the overall signal-intensity, which reflects tissue composition or pathology. These features are derived from the histogram of voxel intensities and include metrics such as mean, median, minimum, maximum, energy, and entropy. Clinically, first-order statistics such as mean and maximum values are already in use, for example, in the evaluation of native T1 mapping and ECV. Kurtosis, which measures the peakedness of the intensity distribution, reflects the degree of concentration of voxel intensities around the mean, with high kurtosis suggesting increased tissue homogeneity (e.g., a uniformly fibrotic scar). Energy represents the magnitude of the overall signal intensity, as observed in hyper-enhanced regions on LGE imaging. Conversely, entropy quantifies the randomness of voxel intensities and may indicate greater heterogeneity, such as in patchy or heterogeneous fibrosis. Texture features represent a higher-order statistical analysis of the spatial relationships and patterns of voxel intensities within the ROI. These features are capable of quantifying tissue heterogeneity, detecting microstructural abnormalities, and providing potential prognostic biomarkers (e.g., tissue heterogeneity as a predictor of arrhythmic risk in myocardial scar). The most commonly utilized texture matrices in radiomics include the Gray Level Co-occurrence Matrix (GLCM), Gray Level Run Length Matrix (GLRLM), Gray Level Size Zone Matrix (GLSZM), Neighboring Gray Tone Difference Matrix (NGTDM), and Gray Level Dependence Matrix (GLDM) (21). GLCM characterizes the spatial relationship between pairs of voxels with specific gray-level values, providing insight into local tissue heterogeneity (e.g., fibrosis distribution on LGE). GLRLM quantifies the length of contiguous runs of voxels with identical gray-level intensities, useful for distinguishing patterns of fibrosis. GLSZM measures the size of homogeneous zones of voxels with the same intensity, allowing differentiation between homogeneous and scattered fibrotic scarring. NGTDM evaluates the difference in gray levels between each voxel and its neighbors, capturing information on tissue texture such as smoothness or coarseness. GLDM assesses the degree of dependency between gray levels of neighboring voxels, which may reflect texture complexity in diseased myocardium.

**Table 1:** Description of radiomic features families and utility in CMR.

Family	Description	Features	Examples of Applications in CMR
First Order	Quantify distribution of voxel signal-intensity through commonly used and basic metrics.	10 percentile, 90 percentile, energy, entropy, interquartile range, kurtosis, maximum, mean absolute deviation, mean, median, minimum, range, robust mean absolute deviation, root mean squared, skewness, total energy, uniformity, variance	Reflection of tissue composition, including fibrosis, myocardial hypertrophy, and nuclear degeneration
Shape (2D + 3D)	Features independent of the gray level intensity distribution in the ROI. Features are derived from approximate shape defined by triangle mesh (3D) or circumferential mesh (2D).	3D: mesh volume, voxel volume, surface area, surface area volume ratio, compactness, flatness,	Evaluation of myocardial geometry
		2D: mesh surface, pixel surface perimeter, perimeter to surface ratio, Both: sphericity, max diameter, major axis length, minor axis length, elongation	
Gray-level co-occurrence matrix (GLCM)	Describe spatial relationship between pairs of voxels with specific gray-level values	Autocorrelation, cluster prominence, cluster shade, cluster tendency, contrast, correlation, difference average, difference entropy, difference variance, ID (inverse difference), IDM (inverse difference moment), IDMN (inverse difference moment normalized), IDN (inverse difference normalized), IMC1 (informational measure of correlation 1), IMC2 (informational measure of correlation 2), inverse variance, joint average, joint energy, joint entropy, MCC (maximal correlation coefficient), maximum probability, sum average, sum entropy, sum squares	Assessment of spatial texture heterogeneity to help evaluate distribution of fibrosis
Gray-level dependence matrix (GLDM)	Quantify degree of dependency between gray levels of neighboring voxels	dependence entropy, dependence non-uniformity normalized, dependence non-uniformity, dependence variance, gray level non-uniformity, gray level variance, high gray level emphasis, large dependence emphasis, large dependence high gray level emphasis, large dependence low gray level emphasis, low ray level emphasis, small dependence emphasis, small dependence high gray level emphasis, small dependence low gray level emphasis	Characterization of tissue texture/complexity
Gray-level run length matrix (GLRLM)	Quantify length of contiguous runs of voxels with identical gray-level intensities	gray-level non-uniformity normalized, high gray level run emphasis, long run emphasis, long run high gray level emphasis, long run low gray level emphasis, low gray level run emphasis, run entropy, run length non-uniformity normalized, run length non-uniformity, run percentage, run variance, short-run emphasis, short-run high gray level emphasis, short-run low gray level emphasis	Characterize and differentiate patterns of fibrosis
Gray-level size-zone matrix (GLSZM)	Quantify size of gray level zones, i.e., the number of connected voxels that share the same gray level intensity, in an image.	high-gray level zone emphasis, large area emphasis, large area high gray level emphasis, large area low gray level emphasis, low gray level zone emphasis, size zone non-uniformity normalized, size zone non-uniformity, small area emphasis, small area high gray level emphasis, small area low gray level emphasis, zone entropy, zone percentage, zone variance	Differentiation of homogenous vs. scattered areas of myocardial scar or other pathologic involvement
Neighboring gray-tone-difference matrix (NGTDM)	Quantify the difference between a gray value and the average gray value of its neighbors within distance in an image.	busyness, coarseness, complexity, strength	Characterization of tissue microstructures

Descriptions and features summarized from pyRadiomics Documentation (21).

In addition to the features derived from the original images, the application of various image filters can enhance specific image characteristics and facilitate the extraction of additional radiomic features. Commonly applied filters include exponential, gradient, logarithmic, squaring, square-root, local binary pattern, and wavelet transforms. These filters enhance the visibility of structures and patterns that may not be readily discernible in the original image data.

There is increasing interest in using deep learning methods for radiomic feature extraction. Radiomic features derived from first-order statistics, texture analysis, and filtering are engineered from handcrafted, established formula based on expert knowledge; by contrast, deep learning features are derived from the data itself using neural networks without predefined feature engineering. Further review of radiomic analysis with deep learning architecture in CMR is described elsewhere (22).

### 2.3 Basic workflow of radiomic data extraction

**Figure 2:**
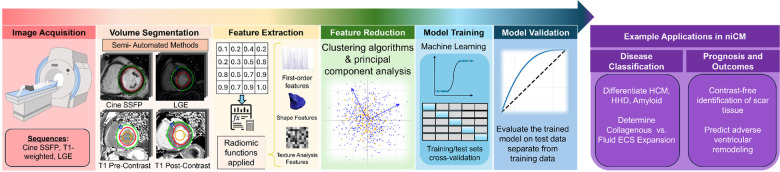
Overview of radiomic workflow and applications. Created in https://BioRender.com.

A standard pipeline to conduct CMR radiomic analysis is illustrated in Figure 2 and includes volume segmentation, feature extraction, feature reduction, model training, and model validation. Volume segmentation involves definition of the ROI via delineation of the myocardium on cine images by tracing the epicardial and endocardial contours, or the segmentation of myocardial scar tissue on LGE images. This segmentation allows for extraction of voxel-level values. Next, quantitative features are extracted, typically categorized into shape, first-order, and texture features. Feature extraction is most commonly performed using available tools such as PyRadiomics (21).

Feature extraction typically yields a high-dimensional dataset, often comprising several hundred features. While this richness provides comprehensive information, it also introduces redundancy and increases the risk of overfitting, as many features may be highly correlated or lack prognostic or predictive relevance. Consequently, feature selection is a critical subsequent step, aimed at identifying the most informative and non-redundant features. This process often begins with redundancy reduction, commonly achieved by eliminating features exhibiting high inter-feature correlation (e.g., Pearson correlation coefficient >0.8). Feature selection methodologies in ML can be broadly categorized into supervised and unsupervised approaches. Supervised techniques select features that are most predictive of a specific outcome variable, leveraging labeled datasets. In contrast, unsupervised techniques focus on reducing dimensionality and redundancy without reference to an outcome variable. Frequently used supervised techniques include random forest classifiers, support vector machines, and eXtreme Gradient Boosting. Common unsupervised approaches include principal component analysis and hierarchical clustering.

## 3 Applications of radiomics and ML in non-ischemic cardiomyopathies

Using ML techniques with radiomic data from CMR has enhanced myocardial tissue characterization in NICM. By extracting quantitative features from standard imaging sequences, these techniques can improve diagnostic accuracy, aid in differential diagnosis, and potentially reduce the need for contrast agents. In this section, we review key studies that have leveraged radiomics and ML across various NICM subtypes. A summary of the referenced studies is provided in Table 2.

**Table 2:** Summary of studies utilizing radiomics in CMR.

First Author (Year)	Scanner/Field Strength	Prediction task	Dataset size (patients)	Sequence	Features extracted (n) | Selected (n)	Feature Reduction Method	ML algorithm of best model	AUC Best Model^a^
Hypertrophic Cardiomyopathy								
Fahmy (2022)	Multi-center/vendor 1.5 T scanners	Screening for myocardial scar in HCM patients	759 internal, 100 external	bSSFP cine^b^	944 radiomic features, 256 deep learning features | 20 total	No reproducibility, fivefold cross-validation; selection by LASSO regression and hyperband optimization.	DL-Radiomics model combining CNN and logistic regression	0.81 (internal), 0.74 (external)
Mancio (2022)	1.5 T scanners (multi-center, multi-vendor)	Screening for myocardial fibrosis in HCM patients	882 internal, 217 external	Cine	2,613 | 7	Reproducibility by ICC^c^ > 0.80; ten-fold cross validation	XGBoost (combined with wall thickness and thickening)	0.83 (internal), 0.83 (external)
Neisius (2019)	1.5 T Philips Achieva	Discriminating HHD^d^ vs. HCM using T1 mapping	232 (53 HHD, 108 HCM, 71 controls)	Native T1 Mapping	152 | 6	Reproducibility by ICC ≥ 0.60; selection by sequential forward selection	SVM^e^ classifier	0.89 (test set)
Pu (2023)	1.5 T scanners (GE Signa Excite HD, Siemens Avanto)	Identifying fibrosis in HCM patients using radiomic features	273 (training: 191, test: 82)	Cine	1,688 | 100	Reproducibility by ICC ≥ 0.85; selection by Boruta algorithm	XGBoost (integrated image + radiomics model ICMR + R2)	0.898 (test set)
Wang (2020)	3.0 T (Siemens Magnetom Skyra)	Discriminating MYH7 and MYBPC3-associated HCM	102 (MYH7: 68, MYBPC3: 34)	T1 Mapping	157 | 157	Reproducibility by ICC; selection by PCA	SVM with PCA	0.886 (test set)
Jiang (2022)	Multiple centers/1.5 T, 3T	Differentiating HCM, DCM, and normal patients using radiomics	283 (48 HCM, 52 DCM, 123 Ctrl)	Cine Imaging	567 | 11	Reproducibility by ICC ≥ 0.80; selection by numerous methods	Random Forest (RF), 5-fold cross-validation|	0.938 for normal controls, 0.966 for DCM, 0.936 for HCM|
Dilated Cardiomyopathy								
Amyar (2025)	3T	Determine association between CMR radiomic features and histological features in patients with non-ischemic cardiomyopathy.	132	Native T1, LGE, ECV maps	1,023 | 6	Reproducibility by ICC > 0.6, selection by Consensus Hierarchical Clustering	–	–
Chang (2023)	3T	Use radiomic features to predict LV remodeling in non-ischemic DCM.	274	Native T1 map	869 | 16	Selection by LASSO regression + Bootstrapping >300 iterations	–	0.811
Nakamori (2023)	3.0 T (Ingenia 3 T, Philips Healthcare)	Differentiate non-collagen vs. mild/moderate collagenous ECS; identify inflammation	132 (DCM)	Native T1, ECV Mapping, LGE	1,023 | 5	Reproducibility by ICC ≥ 0.75; selection by PCA	Logistic Regression with Principal Radiomics	Native T1: 0.75; ECV: 0.79; LGE: 0.74
Cardiac Amyloidosis								
Antonopoulos (2021)	Ingenia 1.5 T MR system (Philips Healthcare)	Classify patients into one of four cardiac phenotypes—normal myocardium, LVH, HCM, or CA	149	T1 Mapping	850 | 84	Reproducibility with CV < 10%; feature reduction by Spearman Correlation eliminating highly correlated features	Random Forest Classifier	0.753
Agibetov et al., 2022	1.5 T MAGNETOM Avanto (Siemens Healthcare)	Distinguish CA from unrelated heart failure types	502 (CA) 420 (control	LGE, MOLLI (T1-mapping), Cine	—	CNN training	CNN (Fine-tubed Model)	0.96 (LGE), 0.93 (MOLLI), 0.91 (CINE)
Shan Huang (2022)	3.0 T MAGNETOM Skyra/Tim Trio (Siemens Healthcare)	Differentiate CA from HCM	100 CA, 217 HCM	T2WI	837 | 7	Reproducibility by ICC ≥ 0.75; selection with Boruta algorithm and LASSO regression	classification tree model	0.842
Shu Jiang et al., 2022	3.0 T MAGNETOM Skyra (Siemens Healthcare)	Differentiate CA vs. HCM	85 CA, 82 HCM	Non-contrast Cine	275 | 19	Reproducibility by ICC ≥ 0.75; selection with LASSO regression	SVM	0.89
Zhou (2022)	3.0 T TIM Trio scanner (Siemens, Germany), 3.0 T MAGNETOM Skyra scanner (Siemens Healthineers, Erlangen, Germany)	Identify CA in patients with known AL amyloidosis	200	LGE	275 | 7–10	Reproducibility by ICC > 0.80; feature selection by Boruta Algorithm	XGBoost classifier (trained on selected features)	0.89
Sarcoid Cardiomyopathy								
Mushari (2024)	An integrated PET/MR system was used to perform simultaneous CMR and [18F]FDG PET (Biograph™ mMR, Siemens Healthcare, Erlangen, Germany)	Differentiate cardiac sarcoidosis vs. myocardial inflammation due to COVID-19	35 post-covid	LGE	1,906 | 2	Spearman Correlation eliminating highly correlated features	Random forest classifier (trained on Composite signature)^f^	Random Forest classifier: AUC 0.91
			40 cardiac sarcoid					
Myocarditis								
Baessler (2019)	1.5T	Distinguish EMB + ^g^from EMB- patients with acute and chronic heart failure-like myocarditis	71	T1, T2 maps	287 | 2	Reproducibility by ICC ≥ 0.75; selection by LASSO regression, Knockoff filter on Random Forest	Random Forest	0.76 (acute), 0.85 (chronic)
Cavallo (2022)	1.5 Tesla, Ingenia, Philips, Best, Netherlands	Prediction of LGE presence from STIR CMR images in acute myocarditis patients	19	STIR	337 | 9	Selection by Weka data mining (v3.8.5)—Correlation-based Feature Selection algorithm	Ensemble ML (EML) combining models	0.79

^a^ From model application to validation cohort.

^b^ bSSFP, balanced steady-state free precision.

^c^ ICC, intraclass correlation coefficient.

^d^ HHD, hypertensive heart disease.

^e^ SVM, support vector machine.

^f^ Numerous other ML models were tested, with Random Forest found to have best performance.

^g^ EMB, endomyocardial biopsy.

### 3.1 Hypertrophic cardiomyopathy and hypertensive heart disease

**Figure 3:**
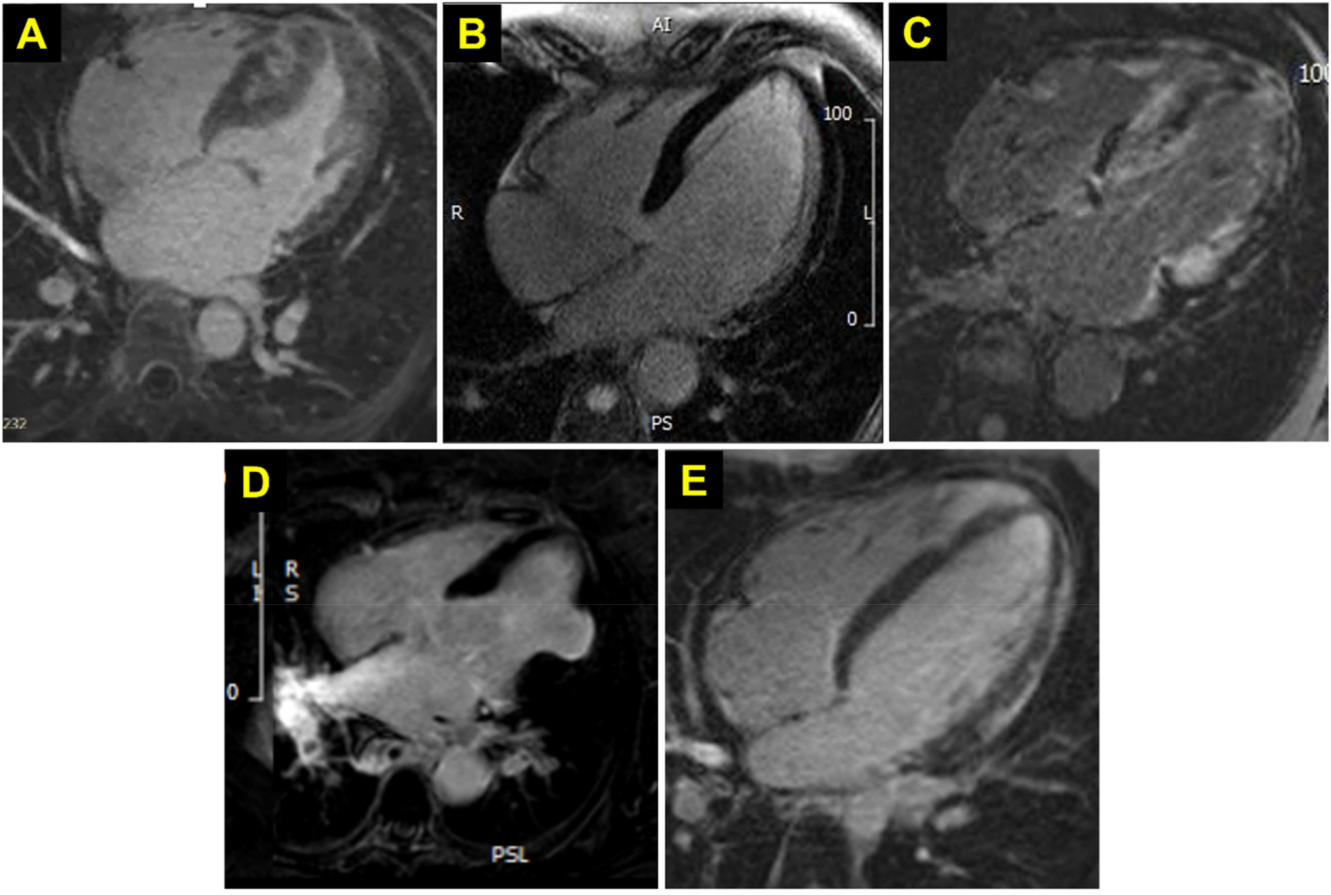
Representative LGE CMR sequences in niCM. Representative LGE four-chamber view images from selected NICM entities. **(A)** hypertrophic cardiomyopathy. **(B)** dilated cardiomyopathy. **(C)** cardiac amyloidosis, **(D)** cardiac sarcoidosis, **(E)** myocarditis.

Familial hypertrophic cardiomyopathy (HCM) is a genetic condition characterized by disorganized LV hypertrophy that can result in LV outflow tract obstruction, as well as myocardial fibrosis that can increase risk of unstable arrhythmia and sudden cardiac death. As a result, patients undergo life-long serial GBCA-enhanced CMR to assess extent of myocardial fibrosis (Figure 3A). AHA/ACC 2024 guidelines recommend GBCA-enhanced CMR imaging every 3–5 years to monitor progressive LGE, as the presence of LGE ≥15% LV mass is associated with a two-fold increase in SCD risk. Thus, accurate and serial assessment of LGE is an important factor in patient guidance, prognosis, and the decision to place an implantable cardiac defibrillator (23). While GBCA are largely safe and well-tolerated, patients with HCM may receive cumulative greater exposure through repeat CMR. Though any risk of recurrent GBCA exposure is unclear, cautionary practices may minimize contrast exposure (24).

Recent studies have shown promise in using ML algorithms to evaluate radiomic features extracted from non-contrast cine images to predict myocardial fibrosis in the absence of contrast. One such approach in HCM combined both conventional standard feature extraction and ML modeling techniques with convolutional-neural network (CNN)-based feature extraction and modeling (25). This combined approach showed acceptable performance and outperformed alternate models based solely on radiomics or deep learning alone (AUC 0.74). Additionally, an ML model using radiomic features extracted from cine images of HCM was able to predict and exclude the presence of myocardial fibrosis with an AUC of 0.83, which may reduce the frequency of routine GBCA administration (26). Further, Pu et al. developed multiple models to assess for myocardial fibrosis in HCM (27), one using predictive image features from CMR (including maximal wall thickness, LV ejection fraction, and left atrial diameter), another using radiomic features derived from the whole LV myocardium. On their testing, it was a model combing both traditional CMR image features with radiomic features that performed the best and with excellent diagnostic performance (AUC 0.898). These studies together support the potential for ML algorithms applied to radiomic data from non-contrast CMR cine sequences to reduce the need for GBCA-enhanced exams to predict myocardial fibrosis in patients with HCM.

Hypertensive heart disease, characterized by concentric myocardial hypertrophy in response to prolonged afterload on the LV, can be difficult to distinguish clinically from HCM, as both may have increased LV wall thickness and myocardial fibrosis. ML algorithms to evaluate radiomic features from native T1 mapping CMR native radiomics features from T1 mapping offer the potential to distinguish these conditions based on subtle differences in myocardial texture. Work by Neisius et al. produced an excellent model trained on 6 out of 152 radiomic features extracted from native T1 maps, which include 2 run-length matrix features, short run high gray-level emphasis and 4 local binary pattern histogram indices to distinguish these conditions, outperforming the predictive ability of global native T1 (e.g., average T1 relaxation time across the ROI) as a surrogate marker for fibrosis (AUC 0.89) (28).

### 3.2 Dilated cardiomyopathy

DCM is a heterogeneous condition with a range of underlying tissue abnormalities that contribute to expansion of the extracellular space (ECS). Histologically, they can be categorized into collagenous ECS expansion due to reactive myocardial fibrosis and collagen deposition and non-collagenous matrix deposition due to inflammation or myocardial edema. ECV-mapping, in which gadolinium contrast is used to extend the standard T1 mapping sequence, has emerged as a promising modality to quantify extracellular expansion (Figure 3B). This technique, however, is limited in its ability to discern the underlying histologic phenotype responsible for observed ECS expansion. Addressing this gap in diagnostic precision is critical to determine the appropriate therapeutic strategy, as identifying inflammatory changes early could enable timely therapeutic interventions prior to myocardial fibrosis. To address this, there has been growing interest in using radiomics to facilitate non-invasive, advanced tissue characterization in DCM patients. Recent work from Nakamori et al. describe a logistic regression approach, trained on radiomic features, capable of distinguishing collagenous vs. non-collagenous ECS expansion across native T1, ECV-mapping, and LGE sequences (29). Expanding on this work, in a study of patients with DCM who received endomyocardial biopsy, radiomics analysis of native T1, ECV, and LGE were associated with histopathologic features including fibrosis, inflammation, myocardial hypertrophy, and fat replacement (30). Together, these studies raise excitement for the power of using radiomic signatures to non-invasively capture the information to differentiate histologic phenotypes in DCM and guide treatment. While analysis of radiomics features may assist in guiding treatment, it also holds promise in prognosticating treatment response in DCM. A single center study of patients with DCM showed that models containing clinical data and radiomics features from T1 mapping improved upon those using clinical data and LGE data (AUC 0.794 vs. 0.716), and best when all data were combined (AUC 0.811) (31). Expansion of radiomics across DCM may allow for improved diagnosis and monitoring in DCM and may guide individualized therapy for patients in the future.

### 3.3 Inflammatory and infiltrative cardiomyopathies

Inflammatory and infiltrative cardiomyopathies present unique diagnostic challenges due to overlapping clinical and imaging features with other cardiac and systemic conditions. As with other cardiomyopathies, radiomics has shown potential for enhancing the precision and accuracy of tissue characterization in these complex diseases.

#### 3.3.1 Cardiac amyloidosis

Cardiac amyloidosis (CA) is a restrictive cardiomyopathy secondary to the extracellular deposition of amyloid fibrils in the myocardium that results in progressive cardiac complications, including diastolic dysfunction and heart failure. Its incidence is higher than previously thought, with estimates between 13%–30% of all cases of heart failure with preserved ejection fraction attributable to ATTR amyloid (32). As newer and effective therapies emerge for this condition, the early and accurate diagnosis is critical to improving outcomes. This is made challenging by the numerous comorbid conditions that could otherwise account for patient symptoms, cardiac biomarkers, and the presence of myocardial hypertrophy. While biopsy remains the gold standard for diagnosis, advances in non-invasive imaging techniques, including cardiac scintigraphy imaging (e.g., Tc-PYP and Tc-HMDP scans) and GBCA-enhanced CMR (Figure 3C) have emerged as reliable modalities for diagnosing CA and reduced the need for biopsy (33). Conventional visual analysis of CMR can lead to underdiagnosis of this disease, particularly in patients with early-stage amyloid infiltration without significant LGE on conventional imaging. Several studies have applied radiomics and ML techniques to improve the detection of CA through various CMR imaging sequences. Application of an unsupervised learning approach to radiomic profiles derived from T1 mapping demonstrated that this approach could naturally classify patients into four cardiac phenotypes: normal myocardium, hypertensive heart disease, HCM, and CA, without prior labeling (34). Building on these principles, a support vector machine-based ML classifier trained on radiomic features to distinguish between CA and HCM on non-contrast cine CMR images (14) demonstrated excellent diagnostic performance, outperforming models trained on conventional CMR image metrics such as maximum LV wall thickness, asymmetric septal hypertrophy, LV mass, and LGE presence (AUC 0.89).

T2 weighted models have also been assessed in the diagnosis of CA. T2 mapping, like T1 mapping, creates a pixelwise map of T2 relaxation times, which is a function of excess water content in the tissue. Traditionally, this is clinically useful in the detection of edema in myocardial inflammation. Work from Huang et al. demonstrated an ML model on radiomic features from T2-weighted mapping that effectively differentiated between CA and HCM (AUC 0.84) (35). Extending these efforts, CNN trained on imaging data alone were compared to a pre-trained CNN refined by imaging data and a logistic regression classifier trained on the output of this pre-trained CNN (12), demonstrating highest performance in the fine-tuned CNN model (AUC 0.91–0.96). Collectively, these studies demonstrate the opportunity that radiomics and ML techniques provide to enhance the diagnostic accuracy of CA at earlier stages across various CMR modalities.

#### 3.3.2 Cardiac sarcoidosis

Cardiac sarcoidosis is an infiltrative granulomatous disease that can lead to heart failure, arrhythmia, and sudden cardiac death. Presently, [18F]FDG PET and GBCA-enhanced CMR (Figure 3D) are the primary non-invasive diagnostic modalities for cardiac sarcoidosis. FDG-PET is useful to determine patients who may be candidates for immunosuppressive therapy and additionally identifies extracardiac inflammation that would support use of immunosuppression. In conjunction, CMR to evaluate for morphologic evaluation and pattern of LGE aids in solidifying the diagnosis of cardiac sarcoidosis. Some prevalent patterns of LGE in cardiac sarcoid include multifocal enhancement, direct extension between ventricles via the interventricular septum, subepicardial, and mid-myocardial LGE. PET and CMR, however, are limited in their precision for the diagnosis. [18F]FDG PET detects myocardial inflammation, regardless of etiology, thus cannot distinguish sarcoid from other inflammatory conditions, like myocarditis. Similarly, LGE is a feature of many ischemic and non-ischemic cardiomyopathies and the LGE presentation of cardiac sarcoid may appear non-specific. Moreover, while CMR is sensitive for fibrosis, it is limited in the detection of early disease prior to fibrotic degeneration. Expanding the role of radiomic analysis to improve non-invasive diagnosis of cardiac sarcoidosis, Mushari et al. demonstrated that a supervised ML algorithm trained on radiomics features were able to differentiate patients with post-COVID as compared with sarcoid myocarditis (AUC 0.9) (13). Further such work may further validate and expand the use of radiomics to assess sarcoid myocarditis.

#### 3.3.3 Myocarditis

Radiographic features of myocarditis on CMR include evidence of edema, inflammatory hyperemia, and LGE suggesting scar. In 2009, the Consensus Criteria for Cardiovascular Magnetic Resonance in Myocardial Inflammation developed the Lake Louise Criteria, which focused on these characteristics derived from T2 weighted, early gadolinium enhancement and LGE from CMR images (Figure 3E). In 2018, updates were made to these criteria to incorporate T1 and T2 mapping, improving the sensitivity and specificity. Recent work incorporating ML algorithms has furthered the ability to differentiate myocarditis by CMR. Work from Baessler et al. describe a random forest classifier trained on radiomic features extracted from both T1 and T2 mapping from a group of patients with suspected acute and chronic myocarditis (16). This model could predict the result of biopsy and could outperform conventional methods like the Lake Louise Criteria in the diagnosis of myocarditis. Subsequently, Cavallo et al. reported a radiomic approach for predicting the presence of LGE in patients with myocarditis, using CMR imaging obtained using the short tau inversion recovery (STIR) sequence, a T1-weighted imaging technique that suppresses fat signals to enhance visualization of myocardial edema without the use of contrast (36). Using an ensemble ML model, combining results from multiple ML classifiers, they developed a model that had a strong ability to predict LGE from contrast-free imaging sequences (AUC 0.79), potentially reducing the reliance on GBCA in myocarditis evaluation.

## 4 Radiomics in genotyping and prognostication

Further the additional integration of genotyping with radiomics has the potential to open new avenues in genetic prediction. One analysis demonstrated that radiomic analysis of native T1 mapping images could differentiate between MYH7 and MYBPC3 mutations HCM with high accuracy (AUC 0.88–0.90), suggesting CMR imaging capabilities beyond myocardial structure, function, and characterization and extending to genetic implications (15). The addition of radiomic texture analysis to predictive models of cardiovascular disease has also been shown to improve accuracy. Pujadas et al. compared models utilizing vascular risk factors, standard CMR features, and extracted radiomics features in various combinations to predict incident cardiovascular events, including atrial fibrillation, heart failure, and myocardial infarction (37); their analysis showed the addition of radiomic features to the model provided incremental predictive value over vascular risk factors and CMR indices. As radiomics research continues to identify imaging biomarkers associated with disease progression and outcomes, we foresee the evolution of more personalized treatment strategies. We also foresee that radiomic features may also assist in predicting both therapeutic and adverse reactions to medication, which may help guide clinicians towards therapeutic interventions optimized for individual patient risk profiles.

## 5 Challenges to radiomics analysis and implementation

The field of radiomics in CMR has demonstrated remarkable potential for improving the evaluation and characterization of cardiac pathologies, particularly NICM. However, several challenges hinder widespread utilization, namely its interpretability, reproducibility, and generalizability.

First, interpretation of radiomic outputs and relating it to tissue pathology is nuanced. On a conceptual level, radiomic analysis characterizes the distribution and pattern of signal intensity within a segmented ROI and assumes that these patterns reflect some intrinsic nature of the tissue. With the improvement of this feature extraction and ML algorithms for this analysis, the goal is to generate radiomic signatures via unique patterns of signal intensity that can differentiate cardiac disease states and offer prognostic value. Inherent to this is the conversion of the qualitative image analysis to a mathematic quantification of imaging values that are harder to intuitively link to known tissue-level processes. This creates a kind of “black-box” effect as a model can make predictions based on features the clinician may not perceive and may create new challenges in interpretation.

Next, there are multiple ongoing challenges to reproducibility. While many of the studies reviewed herein are promising, variability in image acquisition protocols, scanner hardware, and reconstruction algorithms can introduce substantial heterogeneity in image quality and subsequently affect the reproducibility of extracted radiomic features and the generalizability of ML models. Establishing standardized imaging protocols, feature extraction methods, and analytical pipelines is essential before radiomics can reliably be brought to the clinical setting. The Image Biomarker Standardization Initiative has been a notable effort to establish guidelines for the standardization of radiomic feature definitions and extraction methods, promoting reproducibility and comparability across different research groups (38).

Lastly, most studies use relatively small, single-center datasets, limiting the generalizability of findings. Developing radiomics models for use in practice requires access to large, diverse datasets. However, data scarcity and patient privacy considerations necessarily constrain the availability and sharing of CMR data. Collaborative approaches are essential to overcome these challenges and enhance the generalizability of radiomics models. Large-scale databases like the UK Biobank, which includes imaging and clinical data from over 100,000 participants, have created extraordinary opportunities for cardiovascular radiomics research (39). Meanwhile, federated learning has emerged as an alternative solution to address data scarcity and privacy concerns in multi-center studies. In principle, federated learning allows ML models to be trained across multiple decentralized institutions holding local datasets, without the need to transfer or share raw patient data. While this solution has shown potential in cardiovascular radiomics (40), federated learning continues to draw concerns, particularly regarding coordination, expense, and handling of data heterogeneity (41, 42). Ultimately, multicenter collaborations can be expected to play a pivotal role in strengthening standardization efforts by fostering consensus on data formats, feature definitions, and validation methodologies.

## 6 Prospective studies, clinical integration, and policy concerns

The majority of radiomics studies in cardiology, including many reviewed here, have been retrospective and limited to small, single-center cohorts. Going forward, large-scale, prospective studies and randomized controlled trials should evaluate the impact of radiomics on clinical outcomes, cost-effectiveness, and patient quality of life. Such research will be essential in the eventual integration of radiomics into modern clinical practice.

However, integration of ML models into clinical practice raises several policy level, ethical, and regulatory considerations, which have increasingly fallen on the shoulders of regulatory bodies such as the Food and Drug Administration (FDA). The FDA has sought to reimagine a total product lifecycle approach for regulating AI and ML-based software as medical devices (43). This regulatory framework has sought to addressing health disparities by emphasizing good ML practices during premarket review and ongoing performance monitoring to mitigate group harms, however critics have raised concerns that these practices do not sufficiently address the potential for AI and ML models to perpetuate or exacerbate existing health disparities (44). Meanwhile, concerns regarding bias and transparency have been voiced among practitioners, particularly in radiology, where reliance on AI algorithms raises challenges such as failures in edge cases and the opacity of decision-making processes (45), highlighting potential gaps in current regulatory frameworks. Recently, the FDA has voiced the need for a harmonized global regulatory approach and advocated for transparent, risk-based regulatory strategies tailored to the complexity of AI systems, underscoring an ongoing effort to balance innovation and safety (46).

## 7 Potential future directions

To address challenges, several solutions have been proposed. Standardization of imaging protocols and harmonization of data processing pipelines are crucial steps in reducing variability and improving reproducibility. Techniques such as ComBat or ML-based normalization can mitigate biases introduced by differences in scanners and protocols. Collaborative efforts to create large, open-access CMR datasets with standardized annotations are essential for enabling robust training and validation of radiomics models. Federated learning offers an innovative approach to facilitate multi-center collaborations while preserving data privacy. Advances in explainable AI can improve the interpretability of radiomic features, allowing clinicians to better understand their relevance to cardiac pathophysiology. Combining radiomics with other data sources, such as proteomics and genomics, could further enhance its clinical utility. Moreover, tissue-based pathologic validation would allow for a deeper understanding of the relationship between radiomics features and histopathological disease correlates. Streamlining regulatory pathways for AI-based tools and fostering collaborations between academia, industry, and regulatory bodies would accelerate the translation of radiomics into clinical practice. Moreover, education and training programs for clinicians and radiologists on radiomic principles and applications can increase acceptance and integration of these tools into workflows. Development of user-friendly software platforms for automated feature extraction would further ease their adoption. Looking ahead, the integration of radiomics with other advanced imaging techniques, such as strain imaging and T1/T2 mapping, as well as multi-omics data, presents exciting opportunities to provide a more comprehensive understanding of cardiac pathophysiology. Radiomics has the potential to identify subclinical myocardial changes before overt disease manifests, paving the way for earlier interventions and personalized therapies. Future efforts should also focus on leveraging radiomics for prognostication and therapy monitoring, particularly in conditions such as HCM and CA. Advances in computational efficiency and cloud-based processing could enable real-time radiomic analysis, providing immediate insights during clinical encounters. Ultimately, fostering interdisciplinary collaboration between clinicians, data scientists, and imaging physicists will be critical to addressing the multifaceted challenges of radiomics and ensuring its clinical relevance. By addressing current limitations and embracing innovative solutions, radiomics in CMR can become a transformative tool for advancing precision cardiology.
